# Supplementary material for: DOT: Gene-set analysis by combining decorrelated association statistics
Source: PLoS Comput Biol. 2020 Apr 14;16(4):e1007819. doi: 10.1371/journal.pcbi.1007819 (PMC7182280; doi:10.1371/journal.pcbi.1007819)
Supplement: Attachment — Submitted filename: PlosComBioRepot.pdf [file pcbi.1007819.s001.pdf]

In this manuscript, the authors combined single-SNP summary statistics in order to conduct joint analysis of a set of SNPs without accessing original genotype-phenotype datasets. To develop efficient overall summary-statistic, the authors used a decorrelation trick To simplify the correlation structure of the the vector of the single-SNP summary-statistics. The later are correlated by construction. Thus, by rotating the this vector over the eigenvectors of its corresponding correlation matrix one can simplify its correlation structure. Although the decorrelation-trick of a response vector is not a new concept—it has been used for kinship matrix several times in linear mixed models in presence of familial data, e.g. FastLMM— the theoretical and analytical development of the DOT p-values in this manuscript is relevant, in the context summary-statistic association,.

Here are some comments for this manuscript:

Major comments:

1- The authors need to be clear that what they propose is combination of single-SNP summary-statistics. There are many other summary-statistic methods that deal with gene-based summary-statistics across different datasets or meta-analysis. If their method can deal with gene-based summary-statistics, then the authors need to make this clear in the manuscript.

2- In the second paragraph of Introduction, the sentence "The correlation among association test statistics for individual SNPs…". This is not true in general. Although the test statistics are functions of the genotype vectors, they may reflect the LD only if the genotype-phenotype relationship is linear. This is the way we assume our models, however, the biology may reveal more complex relationship than linearity.

3- In Scenario 1, 2, more details (detailed steps) are needed on how the datasets were generated.

4- Conclusions from results of scenario 1 and 2 are a bit confusing. The authors claimed that the power may decrease for DOT in presence of homogeneous effect sizes. This is illustrated in scenario 1. However, in Scenario 2, the effect sizes are the same as scenario 1, but DOT has large power. This means that the power loss due to effect size homogeneity is compensated buy heterogeneity of LD. What is exactly the relationship between heterogeneity in effect sizes and in LD?

5- Loss of power in scenario 1 could be also a result of high correlation. A moderate coefficient $\rho = 0.3$ could illustrate if this the case.

6- For a Chi-squares tests, usually the power increases with increasing of the non centrality parameter. In all Tables scenarios, this parameter decreased with increased power and increased L. More explanation needed.

7- Multivariate normality is a strong assumption in multivariate statistics, and even if the summary-statistics are transformed using rank-based inverse normal scores (one of the best transformations), none can guarantee the vector is distributed following multivariate normal distribution. It would be nice to misspecify multivariate normality assumption of the vector of summary-siatistics and conduct type 1 error evaluation as a sensitivity analysis. To misspecify the multivariate normality one can simulate data form a Clayton/Gumbel copula and run the DOT method.

8- Page 12: More details on how the equation (2) is obtained would be appreciated by audience.

9- Page 12: the authors claimed that equation (2) is valid outside the linear model settings. It would be nice if the authors show a scenario when ϵ is not normally-distributed It is not clear how the authors

10- Page 12: The authors claimed that the method can be used in the case of binary outcome and one can used logistic regression model. After that, they argued that "if the error terms ϵ are assumed to be normally-distributed…". To our knowledge, there is no ϵ in the logistic regression model.

11- Page 12: The delta method uses first-order Taylor expansion of a function of a r. v. around it mean. Would the authors give clear details about their delta method in equations (4) and (5)?

12- It is not clear why the authors needed the invariance property. No matter what is the decomposition of R, the DOT test statistic $X^\top X = Y^\top R^{-1}Y$. This is the Mahalanobis distance of Y to 0_L which takes into account the variances and the covariances between entries of Y. This statistic will always be equal to $Y^\top R^{-1}Y$ and does not depend on the decomposition of the correlation matrix.

13- Did the authors develop the theoretical calculation for the case of unstructured correlation matrix? Several approximations and exact calculation are developed in the literature for p-value calculation of quadratic forms of a normal vector. Not clear what the authors did exactly when the correlation matrix does not have a compound-symmetry form.

14- Equation (7): the upper script 2 of a vector line is confusing. Also, why the authors choose $\gamma = \mu^\top E\sqrt{1/\lambda I}$ for the TQ statistic. The following centrality vector parameter $\gamma = \mu^\top E\sqrt{1/\lambda E} = \mu^\top H$ will work also.

15- Page 14: more details on how the method can be realized for the RTP test would be appreciated.

Minor comments:

1- The denominator of coefficient r_{ij} is $\sqrt{p_i(1-p_i)p_j(1-p_j)}$

2- Forth paragraph in the Intro: In VEGAS, what was the variance-covariance matrix in the Monte Carlo procedure described in this paragraph.

3- Page 12: what is the l in the expression of d.

4- Maybe one can start with Type 1 error evaluation before discussing power evaluation.
