# Supplementary material for: DOT: Gene-set analysis by combining decorrelated association statistics
Source: PLoS Comput Biol. 2020 Apr 14;16(4):e1007819. doi: 10.1371/journal.pcbi.1007819 (PMC7182280; doi:10.1371/journal.pcbi.1007819)
Supplement: Attachment — Submitted filename: response_to_reviewers.pdf [file pcbi.1007819.s002.pdf]

REVIEWERS COMMENTS AND OUR RESPONSES
PCOMPBIOL-D-19-01433 - PLOS Computational Biology
DOT: Gene-set analysis by combining decorrelated association statistics

Thank you for the opportunity to revise this manuscript for the PLOS Computational Biology journal. Below we provide an overview of our responses to each reviewer comment.

Reviewer's 1 comments

In this paper, the authors present a new summary-statistics-based method for testing a group of common SNPs in aggregate for association to a phenotype. Unlike previous approaches, the authors' test statistic explicitly (and exactly) removes correlation between the individual SNPs' summary statistics.

I generally like this paper and appreciate the authors' precision and rigor in deriving and presenting their method. Their theoretical results concerning the power of their test as well as others are also a valuable contribution. So I generally feel this is a very solid contribution to the field. In the long-term I would suggest that the authors consider applications of their framework beyond set-testing since my impression is that the growing number of highly significant associations between *individual* SNPs and phenotypes will eventually cause set-testing to decline as an approach in the common-variant realm. But this is beyond the scope of this paper and for now there remains a substantial community of users of set tests who could benefit from the approach described by the authors.

Major comments

Regarding the technical substance of the paper, I have the following major comments:

R1.1 I'm unclear on the phenomenon whereby TQ tests don't experience an increase in power as more SNPs are added to the model, e.g., in Setting 1. Looking at the authors' model, in which the variance of the environmental noise, epsilon, is set at 1, it would seem that the more SNPs I add to the model with non-trivial effects, the more phenotypic variance is produced by the genetics. In the limit of infinite SNPs and constant-magnitude environmental noise then, the phenotype should be deterministically set by genotype. It would seem unintuitive that in this situation the TQ tests wouldn't have full power. What am I missing? Are the authors scaling something somewhere?

We thank Reviewer #1 for the opportunity to clarify the ceiling property of the TQ test. In multiple linear regression, as the number of non-trivial predictors increases, the coefficient of multiple correlation, R^2, increases as well, and eventually approaches 1. It is important to remember that TQ test is not a multiple regression model but a combination of individual simple linear regression results. It is intuitively clear that a combination of multiple coefficients of determination will not be equal to an R^2 from a multiple linear regression model. That said, the TQ test can certainly attain full power if one were to increase sample size, while our simulation results that illustrate TQ ceiling property of the non-centrality parameter (Tables 1-4) were obtained under constant N times the standardized effect size.

To clarify the ceiling property of the non-centrality parameter of the TQ test, we added the following discussion to Scenario 1 setting:

"Further, the table confirms that the decorrelation method is under-performing relative to TQ if there is very little heterogeneity among effect sizes. However, power of all methods would increase under lower correlation. For example, for $\rho = 0.3$ and $L = 20$, the powers for TQ and DOT become 0.98 and 0.67, respectively. Additional insight into power behavior of methods under this scenario can be gained by examining Eq. 19. The asymptotic power for TQ can be simply computed in R as:
`1-pchisq(qchisq(1-0.05, df=1), df=1, ncp=2.35^2/0.7)`.
This gives 0.802 TQ power as $L \to \infty$ for Table 1 and 0.99 for the situation when ρ is lowered to 0.3. This simple approximation is surprisingly precise and works well for the rest of the settings. Scenario 1 is admittedly unrealistic in practice [...]"

R1.2 Relatedly, it would help if the authors included in their methods section more detailed descriptions of their simulation set ups especially including sample size and proportion of phenotypic variance explained by genotype for each simulation (including the simulations with real genotypes).

In our simulation experiments, data were generated in two different ways: (1) we generated individual-level data from real genotypes, performed L simple regression analyses, combined L summary statistics using TQ, ACAT and DOT approaches; (2) simulated L summary statistics directly (completely omitting generation of individual-level data) and combined summary statistics using TQ, ACAT and DOT. We further showed equivalence between generating sections of genome with individual-level data and generating statistics directly (e.g., note the equivalence of values in Table 5 columns "Theor" and "Regr"). Since generating statistics directly substantially decreases computational burden of simulations, we proceed to ***generation of statistics only*** *for the results presented in* ***Tables 1-4****. When statistics are generated directly, the sample size is confounded with the standardized effect size.*

Description of simulations with real genotypes is summarized in ***"LD patterns from the 1000 Genome Project"*** *section as follows:*

"Each stretch of consecutive SNPs contained from 10 to 200 SNPs with the minimum allele frequency 0.025. A random portion of SNPs in every set carried no effect on the outcome on its own, and we considered these SNPs to be "proxies" for causal variants due to LD. The median LD correlation varied from approximately -0.6 to 0.98 between random stretches of SNPs. The number of proxy SNPs varied from 3 to 197 across simulations. The sample size was also set to be random and varied from 500 to 3000 across simulations. Effect sizes for causal variants were modeled by β-coefficients, as given by Eq. (1), and drawn randomly from the interval [-0.4, 0.4]."

To clarify the possible range of phenotypic variance values explained by genotype, we now added the following sentence to the ***"LD patterns from the 1000 Genome Project"*** *section:*

"Different combinations of sample size, the number of causal SNPs, their individual effect sizes and LD patterns among them, resulted in total proportion of phenotypic variance explained (i.e., the multiple correlation coefficient) varying from $10^{-5}\%$ (fifth percentile) to 7% (ninety-fifth percentile) with the mean value of 2.5% and the median value of 1%."

The results of ***simulations with real genotypes*** *are presented in* ***Table 5****, columns labeled* ***"Regr. TQ"*** *and* ***"Regr. DOT."*** *Note that in this table, the power is averaged over all parameter combinations described above to give the reader a fuller understanding of TQ and*

DOT power comparison under most general settings.

R1.3 I don't know if the proportion of variance explained by genotype is high in the authors' simulations. But if it is, do they expect their results to generalize to settings where this is not the case? For real traits, any one set of tens to hundreds of contiguous SNPs typically only explains a very small proportion (on the order of 1%, usually even less than that) of phenotypic variance, so I'd be interested to see if this is the case in the simulations here. Sometimes it's okay to simulate small sections of genome explaining high proportions of phenotypic variance as long as sample size is lowered in some corresponding way, but if this is the case here the authors should explain and perhaps use their theory to justify.

*In response to **R1.2**, we note that we generated data in two different ways and would like to re-emphasize that the two approaches were shown to be equivalent.*

Statistical power is the product of $\sqrt{N}$ and the proportion of phenotypic variance explained (or the correlation between Y and SNP_j). You correctly point out that the same power can be attained with (1) a small sample size and a SNP that explains relatively high proportion of phenotypic variance or (2) a large sample size and SNP that explains relatively small proportion of phenotypic variance. However, because the proportion of phenotypic variance explained is confounded with the square root of the sample size and standardized effect size, our results in Tables 1-4 are generalizable to either scenario, i.e., the same test statistic can be obtained when a SNP explains high proportions of phenotypic variance but the sample size is low or a SNP explains low proportion of phenotypic variance but the sample size is high.

R1.4 How do the authors expect their statistic to behave in the presence of near-perfect LD? It seems they don't regularize their LD matrix, which surprised me. I would be interested to see power results under a simulation setting where two SNPs, only one of which is causal and contains 75% of the causal signal in locus, have a) 99% correlation and b) 100% correlation.

We apologize for not making it clear that in the situations of near-perfect LD the correlation matrix among statistics may not be invertible. There are many methods available that will provide a solution to non-invertibility issue. In our case, we used the `nearPD()` *function available in R to make the correlation matrix definite positive. When using* `nearPD()` *during the analysis of real data, we checked the difference between the values in the original correlation matrix and the ones returned after applying this function. The magnitude of the difference was negligible (close to 10^{-3}). We now clarify our solution to non-invertibility and provide additional power simulation results for the scenario that you described above in the first paragraph of the "Results" section. The R script for these additional simulations is also added to the GitHub page accompanying this article.*

R1.5 For the simulations with real genotypes, how was the 100kb region on chromosome 17 chosen? Do the authors expect the simulation results to generalize to other regions of the genome as well? If they are unsure, is it computationally feasible to do simulations where random sets of contiguous SNPs are chosen from the whole genome?

There was no particular reason for us to choose this chromosome but we expect our results to be generalizable to other regions of the genome in the sense that LD structure among SNPs on chromosome 17 is representative of LDs throughout the genome. Perhaps more important,

and a potential limitation of our simulations, is the way we chose the association model. That is, we assumed high heterogeneity in effect sizes and combined statistics of only proxy SNPs. Nonetheless, the analyses of real data (both the original one and the one additionally included; see response to R1.6) also support our conclusion that DOT often has higher statistical power than TQ or ACAT.
We added the above discussion of choosing regions on chromosome 17 to the "LD patterns from the 1000 Genome Project" section.

R1.6 How were the genes *ESR1*, *FGFR2*, *RAD51B*, and *TOX3* chosen by the authors for demonstration of their method? Does this set include all the genes found in the Min et al paper to have association with breast cancer? Would it be possible to test a larger set of genes chosen more systematically so that readers can have a sense for whether the authors' approach should in general be preferred over other approaches? Or perhaps to test a few genes chosen by authors of other set testing methods papers?

We used all the genes found in the Min et al. paper that were also robustly replicated by different research groups.
We are also happy to oblige with your suggestion of testing a few more genes and included additional results for genes studied in connection to cleft lip in the revised version of the manuscript.

R1.7 Do the authors think it would make sense to compare (either in simulation or in practice) to the gene-level test in de Leeuw 2016 PLOS Comp Bio since that method also provides a way to test the SNPs surrounding an individual gene for association while accounting for correlation between variants in order to boost power? Relatedly: ACAT seems to be a method intended primarily for testing of rare variants in sequence data; could it be that this makes it an inappropriate comparison point?

ACAT authors write: "ACAT is a general method for combining p values and can be used in different ways depending on the types of p values being combined" (Liu et al., 2019). The method does not assume that P-values correspond to rare variants; it transforms P-values into Cauchy random variables to deal with dependence among first-order statistics when the number of tests is very large. Therefore, we find this method to be a direct competitor to DOT.
MAGMA method analyzes the summary SNP statistics by considering the mean of the χ^2 statistic for the SNPs in a gene or the top χ^2 statistic among the SNPs in a gene (de Leeuw et al., 2015). The mean of χ^2 statistic is equivalent to Fisher's method for combining dependent P-values or the TQ (Brown, 1975; Hou, 2005). The top χ^2 statistic among the SNPs in a gene is equivalent to the Bonferroni correction for dependent tests. Our recent article provides a detailed comparison of the χ^2 -based methods to the Simes test, which can never perform worse than Bonferroni (https://www.ncbi.nlm.nih.gov/pmc/articles/PMC6879667/).

In the revised manuscript, we now reference the de Leeuw et al. (2015) article in the "Results" section and discuss why we do not include a comparison to it in our simulations.

R1.8 I liked the way the authors argued for their particular choice of pseudoinverse by suggesting that exchangeability of SNPs should be preserved by this operation. Kudos!

We thank the Reviewer for this comment.

Minor comments

R1.1.1 It seems that the claims about the scaling of power as a function of L are for fixed $\rho > 0$, because when $\rho = 0$ the tests considered are equivalent. The authors may want to clarify this.

We concur with your recommendation and added this clarification to the first paragraph of Results section.

R1.1.2 In the definition of r_{ij} on page 2, should there be a square-root in the denominator?

Thank you for catching this typo! It is now fixed.

R1.1.3 On page 3 there is a typo in "This general idea is straightforward and HAVE been used..." (emphasis mine)

Once again, thank you for catching this typo. We fixed it.

R1.1.4 What was the sample size of the breast cancer data set that the authors analyzed?

We added this information. It was 1277 Caucasian triads for the breast cancer data.

R1.1.5 In Equations 4 and 5, ρ_{ij} appears on both sides of the equations.

Thank you. We fixed our notation.

R1.1.6 The derivation of the covariance matrix of the vector of summary statistics can be carried out without the delta method but under the assumption of Gaussian genotypes (which is justifiable for large sample size and MAF bounded away from zero). See Proposition 2 in the supplement of Reshef et al. (2018). The authors may wish to comment on whether these two derivations give different results and if so why not.

We agree that the assumption of Gaussian genotypes can be use. However, both Eqns (22) and (27) in Reshef et al. (2018) derive the first two moments of the summary statistics under the null hypothesis, while we were considering the case under the alternative hypothesis.

R1.1.7 For the results in Table 6: 1) which set of genotypes were the phenotypes simulated from? 2) Which set of genotypes was used as the reference panel? The only genotypes I saw mentioned were 1000 Genomes, but two distinct sets of genotypes are required for the described analysis.

We added the following clarification:

Reference panels for these simulations were obtained as follows. Each LD matrix derived from real data was assumed to represent the population matrix. Next a sample was drawn and the corresponding sample LD matrix, was calculated. That matrix should have been used for calculations of the gene-based test statistics. Instead, we drew a separate sample of size

N , assuming the same population LD matrix. In the calculation of the tests, that sample correlation matrix was used in place of the correct one.

Reviewer's 2 comments

Zaykin et al propose DOT, a new method for Gene Based Association Testing. There is demand for a gene (or set-based) method, so a method that improves upon previous methods would be of much interest and (with easy to use software) could become highly used. Zaykin perform many simulations to show that DOT has the potential to improve on a state-of-the-art method, VEGAS (and also ACAT, a method I am not familiar with). They also have a real data example, but this is very limited. While I am not convinced from this draft alone, I believe that by including an extra simulation method, and a more convincing application, DOT could be a useful addition to the field.

Major comments

R2.1 Reading the method (and apologies that I did not understand all the details), DOT appears similar to methods which first compute principal components for each gene (ie eigen decompose the snp snp correlation matrix), then regress the phenotype on these (consider the following paper, or derivatives https://onlinelibrary.wiley.com/doi/pdf/10.1002/gepi.20219). Thus I require convincing this method is different to / an improvement on those.

Thank you for your comment. We now cite Gauderman et al. (2007) in our Introduction. However, the key difference between PC-analysis that computes linear combination of SNPs in Gauderman et al. (2007) and the methods considered in our manuscript (TQ, ACAT, DOT) is data availability. Gauderman et al. (2007) method requires individual-level data and does not operate on summary statistics.

R2.2 The format of the paper makes it challenging to read. Usually methods would come before results. However, if the journal requires such a style, then you must give some brief details at the start of results.

Here, we ask the editor to give us advice. On the one hand, the journal requires the "Materials and Methods" section to appear after Discussion. On the other hand, as per your request, we can re-structure the order of presentation. Either way, we now provide more detail about our method in the beginning of the Results section.

R2.3 I consider there to be insufficient detail of the simulations. For example, I can't see sample size and rho was hard to find. Is it the case for all simulations that all L snps are assigned effects, or just the first one?

Revisions have been made to elucidate details of our simulation studies. Additionally, please see our responses to R1.1, R1.2, and R1.3.

R2.4 It is good you compare with vegas (TQ?). But to my knowledge, the most common methods are SCAT, or magma, and my preferred is Fast-LMM-Set, so would ideally like at least one of these considered (or a statement with justification that these very similar to VEGAS) ps are assigned effects, or just the first one?

Thank you for your comment. To explain why we do not include SKAT into our power comparisons, we write the following in the beginning of the Results section:

" Specifically, Liu et al. found ACAT to be competitive against popular methods, including SKAT and burden tests for rare-variant associationsWu et al. (2011); Li and Leal (2008); Madsen and Browning (2009); Price et al. (2010)."

In response to your concern and that of Reviewer 1 (please see R1.7), we now also included the discussion of the MAGMA method:

"Among other similar approaches is MAGMA (de Leeuw et al., 2015). MAGMA analyzes summary association statistics by considering the mean of the chi-square statistic for the SNPs in a gene or the largest statistic among the SNPs in a gene. The mean of statistics method is equivalent to Fisher's method for combining dependent P-values (Brown, 1975; Hou, 2005). The method based on the top chi-square statistic among the SNPs in a gene is equivalent to the Bonferroni correction for dependent tests. There have been extensive studies comparing these two methods (Vsevolozhskaya et al., 2019). Note that TQ is very similar to the Fisher method."

Finally, we note that both SKAT and Fast-LMM-Set require individual-level data and can not be directly compared to methods that are based on the combination of summary statistics.

R2.5 I believe you require odds ratios for the SNPs in table 8 (ideally from multi snp analysis and perhaps those from single snp)

The analysis in Table 8 can not provide either an odds ratio or a P-value. The top ranked SNPs were identified by considering the top three components in the linear combination $DOT = \sum_{i=1}^{L} X_i^2$*, where* X_i*'s are the decorrelated summary statistics. Once the highest three values of* X_i^2 *were identified for each gene, we considered individual components of* $X_i = \sum_{j=1}^{L} h_j Z_j$ *that are formed as a linear combination of the original statistics weighted by the elements of matrix* $\mathbf{H}$*. The top individual components* $h_j Z_j$ *(with the same sign as* X_i*) correspond to individual SNPs presented in Table 8.*

Minor comments

R2.1.1 I applaud the range of simulations, and also of considering situations where DOTS is not well-suited.

We thank the Reviewer for this comment.

R2.1.2 I also like the insight into how DOT has the potential to gain power (when a wide spectrum of effect sizes, which is thought likely to be the case with complex traits).

Once again, we appreciate your comment.

R2.1.3 In the simulations, it is hard to understand the effect sizes. Can you instead report in terms of heritability, ideally both (average) phenotypic variance explained by the gene/region, and (average) variance explained by most significant individual snp.

Thank you for this comment. To shed light on the amount of phenotypic variance explained in our simulations, we now write:

"Different combinations of sample size, the number of causal SNPs, their individual effect sizes and LD patterns among them, resulted in total proportion of phenotypic variance explained (i.e., the multiple correlation coefficient) varying from 10^{-5}% (fifth percentile) to 7% (ninety-fifth percentile) with the mean value of 2.5% and the median value of 1%."

Additionally, please see our response to R1.2.

R2.1.4 The tables (and I think figures) require captions. In generally, these should give a full description (or if the same, say "see Table 1... etc"), rather than relying on the user to parse through the main text.

We followed journal formatting quidelines that state: "Tables require a label (e.g., "Table 1") and brief descriptive title to be placed above the table."

R2.1.5 Good that a github page is provided with software (although I have not tested).

Thank you for this comment. With the revised version of the manuscript, we now provide additional R scripts to ensure replicability of our results.

R2.1.6 Please provide a summary of run time for a decent sized analysis.

The run time is nearly instantaneous. We added (last sentence of the first paragraph of Results that "We note that the calculations are very fast and that the 100,000 simulation runs were completed in less than ten minutes on a typical laptop"

R2.1.7 Intro; It is important to distinguish situations ... I suggest you replace second "in which" with "from those" or something similar

Thank you. We replaced the second "in which" appearance.

R2.1.8 I would prefer if you provided more thresholds when testing the false positive rates (e.g. show not just alpha 1e-4, but also say 0.05, maybe a few others, in supplement if necessary)

We added two more tables, $\alpha = 0.001$ *and* $\alpha = 1 \times 10^{-7}$.

R2.1.9 It is good you can accommodate covariates, but is this feature used in application?

No. In data application we were operating on summary statistics obtained from the transmission disequilibrium test (TDT).

Reviewer's 3 comments

In this manuscript, the authors combined single-SNP summary statistics in order to conduct joint analysis of a set of SNPs without accessing original genotype-phenotype datasets. To develop efficient overall summary-statistic, the authors used a decorrelation trick to simplify the correlation structure of the vector of the single-SNP summary-statistics. The later are correlated by construction. Thus, by rotating the this vector over the eigenvectors of its corresponding correlation matrix one can simplify its correlation structure. Although the decorrelation-trick of a response vector is not a new concept – it has been used for kinship matrix several times in linear mixed models in

presence of familial data, e.g. FastLMM – the theoretical and analytical development of the DOT p-values in this manuscript is relevant, in the context summary-statistic association.

Major comments

R3.1 The authors need to be clear that what they propose is combination of single-SNP summary-statistics. There are many other summary-statistic methods that deal with gene-based summary-statistics across different datasets or meta-analysis. If their method can deal with gene-based summary-statistics, then the authors need to make this clear in the manuscript.

Thank you for your comment. In the Introduction of the revised version of the manuscript, we emphasize that we propose a method for combining single-SNP summary statistics by writing that:

"Here, we propose a new decorrelation-based method for combining single-SNP summary association statistics."

Also, the first sentence of the Discussion states:

"In this research, we have proposed a new powerful decorrelation-based approach (DOT) for combining SNP-level summary statistics (or, equivalently, P-values) and derived its theoretical power properties."

R3.2 In the second paragraph of Introduction, the sentence "The correlation among association test statistics for individual SNPs...". This is not true in general. Although the test statistics are functions of the genotype vectors, they may reflect the LD only if the genotype-phenotype relationship is linear. This is the way we assume our models, however, the biology may reveal more complex relationship than linearity.

We thank Reviewer 3 for this comment and fully share this concern. In the revised version of introduction, we add this statement is true only under the assumption of a linear model.

R3.3 In Scenario 1, 2, more details (detailed steps) are needed on how the datasets were generated.

*Revisions have been made to clarify details of data generation. For scenarios 1-4, we generated values of the test statistics directly, which we now clarify in the very beginning of the "**Simulations assuming that the LD matrix and the summary statistics are obtained from the same data.**"*

"To compare methods with and without decorrelation of statistics, we considered several distinct settings. In settings 1-4, the results of each row of the tables were based on one million simulations. Association statistics were simulated directly, namely, a 10^6 by L matrix of MVN vectors was simulated first, and then each row of the matrix was analyzed by the competing methods. The empirical powers were obtained as the proportion of times that a particular statistic value exceeded $\alpha = 0.05$."

Additionally, please see our response to R1.2.

R3.4 Conclusions from results of scenario 1 and 2 are a bit confusing. The authors claimed that the power may decrease for DOT in presence of homogeneous effect sizes. This is illustrated in scenario 1. However, in Scenario 2, the effect sizes are the same as scenario 1, but DOT has

large power. This means that the power loss due to effect size homogeneity is compensated buy heterogeneity of LD. What is exactly the relationship between heterogeneity in effect sizes and in LD?

The insight into why DOT's power increases under this scenario can be gained by contrasting an orthonomal set of eigenvectors of an equicorrelated matrix (Scenario 1) and that of a more general correlation matrix (Scenario 2). The DOT test statistic is formed as a $\sum_{i=1}^{L} X_i^2$, where each $X_i = \sum_{j=1}^{L} w_j Z_j$. In its simplest form, the weights w_j can be represented as the values of the j-th orthonomal eigenvector of the correlation matrix among statistics, $\mathcal{R}$. If $\mathcal{R}$ is equicorrelated (Scenario 1), a number of eigenvectors will have a lot of zero entries and all non-zero entries will sum up to zero. If all Z_j's are similar to one another, under Scenario 1, all $X_i = \sum_{j=1}^{L} w_j Z_j$ will be close to zero due to multiplication by zero or a cancellation of individual terms in a linear combination when a sum is formed. For $\mathcal{R}$ with a more general structure, all w_j's are expected to be more heterogeneous and non-zero, which would increase, on an average, the values of X_i's.

R3.5 Loss of power in scenario 1 could be also a result of high correlation. A moderate coefficient $\rho = 0.3$ could illustrate if this the case.

You are absolutely correct! This conclusion can be attained by examining non-centrality parameter values in Eqs. (13) and (19), which indicate that non-centrality value increases as ρ decreases. The following R code reproduces TQ power in the top row of Table 1 without having to perform simulations:

```
1-pchisq(qchisq(1-0.05, df=1), df=1, ncp = 2.35^2/0.7)
```

Changing the value of ρ from 0.7 to 0.3 will result in higher power. To illustrate this property of the TQ test we added the following discussion to the Scenario 1 section:

"However, power of all methods would increase under lower correlation. For example, for $\rho = 0.3$ and $L = 20$, the powers for TQ and DOT become 0.98 and 0.67, respectively. Additional insight into power behavior of methods under this scenario can be gained by examining equation 19. The asymptotic power for TQ can be simply computed in R as: `1-pchisq(qchisq(1-0.05, df=1), df=1, ncp=2.35^2/0.7)`. This gives 0.802 TQ power as $L \to \infty$ for Table 1 and 0.99 for the situation when ρ is lowered to 0.3. This simple approximation is surprisingly precise and works well for the rest of the settings"

R3.6 For a Chi-squares tests, usually the power increases with increasing of the non centrality parameter. In all Tables scenarios, this parameter decreased with increased power and increased L. More explanation needed.

Although the average non-centrality parameter is decreasing, the number of statistics that we combine is increasing. Thus, it should not be surprising to see gain in power as more results (although weaker) are combined. Also, we report the average non-centrality value, not the sum of all non-centralities or the maximum value.

R3.7 Multivariate normality is a strong assumption in multivariate statistics, and even if the summary-statistics are transformed using rank-based inverse normal scores (one of the best transformations), none can guarantee the vector is distributed following multivariate normal distribution. It would be nice to misspecify multivariate normality assumption of the vector

of summary-siatistics and conduct type 1 error evaluation as a sensitivity analysis. To mis-specify the multivariate normality one can simulate data form a Clayton/Gumbel copula and run the DOT method.

We agree with your concern. To check sensitivity of the methods to mis-specification of the multivariate normality assumption, we added another set of simulations (prior to Scenario 1 in the revised manuscript). We simulated the error terms from a heavy-tailed Laplace distribution with unit variance. Our results reveal that both TQ and DOT are robust to violation of multivariate normality.

R3.8 Page 12: More details on how the equation (2) is obtained would be appreciated by audience.

An intuitive explanation can be gained by considering the case of independent predictors, i.e., $\boldsymbol{\Sigma} = \mathbf{I}_L$*. If both the outcome and the set of predictors are standardized, then* $\frac{\boldsymbol{\Sigma}_j \boldsymbol{\beta}}{\sqrt{\boldsymbol{\beta}'\boldsymbol{\Sigma}\boldsymbol{\beta}+1}} = \frac{\beta_j}{\sqrt{\sum_j \beta_j^2+1}}$*, which is a standardized regression coefficient. We modified the main text to include this intuitive explanation.*

R3.9 Page 12: the authors claimed that equation (2) is valid outside the linear model settings. It would be nice if the authors show a scenario when ϵ is not normally-distributed.

We included an additional set of simulations with error terms following a heavy tail distribution (Laplace distribution). This is described in the first paragraph of Results

R3.10 Page 12: The authors claimed that the method can be used in the case of binary outcome and one can used logistic regression model. After that, they argued that "if the error terms ϵ are assumed to be normally-distributed...". To our knowledge, there is no ϵ in the logistic regression model.

The logistic regression model can be viewed as a linear regression model with an error term, where the outcome is an unobserved latent variable that is subsequently dichotomized around its mean. We mention the latent variable model in that part of the text.

R3.11 Page 12: The delta method uses first-order Taylor expansion of a function of a r. v. around it mean. Would the authors give clear details about their delta method in equations (4) and (5)?

Additional details of our deviations can be found at https://www.biorxiv.org/content/10.1101/2019.12.18.881425v1*. In the revised version of the manuscript, we included this reference to the main text*

R3.12 It is not clear why the authors needed the invariance property. No matter what is the decomposition of R , the DOT test statistic $X^T X = Y^T R^{-1} Y$. This is the Mahalanobis distance of Y to 0_L which takes into account the variances and the covariances between entries of Y. This statistic will always be equal to $Y^T R^{-1} Y$ and does not depend on the decomposition of the correlation matrix.

DOT allows one to decorrelate the set of dependent statistics and then combine them by a variety of methods, including Fisher's combination test. However, if we were to decompose the correlation matrix using Helmert or Cholesky decomposition, the value of combined P-value would depend on the order of statistics. Even in the special case of "equicorrelation" with $\rho = 0$*, i.e., when statistics are independent, the combined P-value may change. This statement is very easy to check by running the R script below:*

```
Helmert.eigenvectors <- function(n) {
   ev <- matrix(0, n,n); ev[,1] <- 1/sqrt(n)
   for(i in 2:n) {
      ev[(1 : (i-1)), i] <- 1/sqrt(i*(i-1))
      ev[i,i] <- -(i-1)/sqrt(i*(i-1))
   }
   ev
}
L <- 5; Sgm <- diag(1,L)
y <- rnorm(L)
for(i in 1:5) {
   (y1 <- sample(y))
   (p <- 1-pchisq((y1 %*% Helmert.eigenvectors(L))^2, df=1))
   if(i==1) cat("Pvalues:", sort(p), "\n\n")
    # Fisher combined P-value
   pv1 <- 1-pchisq(-2*sum(log(p)), df=2*L)
   # sum of 1 df chisquare (only here the order of Y's is irrelevant)
   pv2 <- 1-pchisq(sum(qchisq(1-p, df=1)),df=L)
   cat("Combined:", pv1,pv2, "\n")
}
```

Here is the sample output of the code above:

```
Pvalues: 0.1130726 0.2126045 0.3576062 0.6257086 0.7821626
Combined: 0.362065 0.3890419
Combined: 0.3582541 0.3890419
Combined: 0.3582541 0.3890419
Combined: 0.3805313 0.3890419
Combined: 0.3429328 0.3890419
```

The first column of the combined results uses Fisher's combination test and Helmert decomposition of the correlation matrix. It is clear that combined P-value varies for each instance of shuffled y's. The second column outputs statistics based on the sum of 1 degree-of-freedom chi-squares and Helmert decomposition of the correlation matrix. The combined statistics in the second column are invariant to order. However, if we were to use decomposition proposed in DOT, the results of the Fisher combination test will be invariant to permutation of y's.

R3.13 Did the authors develop the theoretical calculation for the case of unstructured correlation matrix? Several approximations and exact calculation are developed in the literature for p-value calculation of quadratic forms of a normal vector. Not clear what the authors did exactly when the correlation matrix does not have a compound-symmetry form.

Yes, our theoretical power calculations are provided for a general unstructured correlation matrix. Note that although Eqns. (6) and (7) are standard, the elements in $\boldsymbol{\mathcal{R}}$, and therefore the eigenvectors, the eigenvalues, and the non-centralities explicitly depend on the β-coefficients through Eqs. (2) and (5) that we derived. Further, Eq. (19) provides new and accurate power approximation for the TQ method in a case of non-equicorrelation matrix obtained as a low rank perturbation of an equicorrelation matrix.

R3.14 Equation (7): the upper script 2 of a vector line is confusing. Also, why the authors choose $\gamma = \mu^T E\sqrt{1/\lambda}I$ for the TQ statistic. The following centrality vector parameter $\gamma = \mu^T E\sqrt{1/\lambda}E = \mu^T H$ will work also.

Each non-centrality parameter can be written as $\gamma_i = \frac{1}{\lambda_i}(\boldsymbol{\mu}'\mathbf{u}_i)^2$, *where* $\mathbf{u}_i$ *is the ith orthonormal eigenvector of* $\boldsymbol{\mathcal{R}}$. *We now re-write this expression using Hadamard product in matrix notation.*

R3.15 Page 14: more details on how the method can be realized for the RTP test would be appreciated.

We added the following clarification to the main text:

"Since DOT produces a set of independent one degree of freedom chi-squares, to use it with with RTP, one can convert the set of chi-squares to P-values and take the product of the first smallest values, which is the RTP statistic."

Also, similar to the situation described in response to R3.12, if one were to use a different decomposition of the correlation matrix, then take the top k results and combine them via RTP, the combined result will not be invariant to re-ordering of top P-values.

Minor comments

R3.1.1 The denominator of coefficient r_{ij} is $\sqrt{p_i(1-p_i)p_j(1-p_j)}$.

Thank you, we fixed this typo.

R3.1.2 Forth paragraph in the Intro: In VEGAS, what was the variance-covariance matrix in the Monte Carlo procedure described in this paragraph.

In VEGAS, the LD is approximated using an external panel. Then, a parametric bootstrap is performed in which multiple vectors of Z-statistics are simulated from MVN with the estimated LD matrix. The P-value is computed as the proportion of squared and added Z's that exceeded the observed value.

R3.1.3 Page 12: what is the l in the expression of d.

We now clarify in the main text that l is some threshold value for the latent variable.

R3.1.4 Maybe one can start with Type 1 error evaluation before discussing power evaluation.

We chose to present the results starting with power comparisons because we wanted to introduce and highlight power characteristics of methods, in part because, according to the journal format, the section "Materials and Methods", where some discussion takes place, appears after "Results".

References

Brown, M. B. (1975). 400: A method for combining non-independent, one-sided tests of significance. *Biometrics*, pages 987–992.

de Leeuw, C. A., Mooij, J. M., Heskes, T., and Posthuma, D. (2015). MAGMA: generalized gene-set analysis of gwas data. *PLoS computational biology*, 11(4):e1004219.

Gauderman, W. J., Murcray, C., Gilliland, F., and Conti, D. V. (2007). Testing association between disease and multiple snps in a candidate gene. *Genetic Epidemiology: The Official Publication of the International Genetic Epidemiology Society*, 31(5):383–395.

Hou, C.-D. (2005). A simple approximation for the distribution of the weighted combination of non-independent or independent probabilities. *Statistics & probability letters*, 73(2):179–187.

Li, B. and Leal, S. M. (2008). Methods for detecting associations with rare variants for common diseases: application to analysis of sequence data. *Am J Hum Genet*, 83(3):311–321.

Liu, Y., Chen, S., Li, Z., Morrison, A. C., Boerwinkle, E., and Lin, X. (2019). ACAT: A fast and powerful P-value combination method for rare-variant analysis in sequencing studies. *Am J Hum Genet*, 104(3):410–421.

Madsen, B. E. and Browning, S. R. (2009). A groupwise association test for rare mutations using a weighted sum statistic. *PLOS Genetics*, 5(2):e1000384.

Price, A. L., Kryukov, G. V., de Bakker, P. I., Purcell, S. M., Staples, J., Wei, L.-J., and Sunyaev, S. R. (2010). Pooled association tests for rare variants in exon-resequencing studies. *Am J Hum Genet*, 86(6):832–838.

Reshef, Y. A., Finucane, H. K., Kelley, D. R., Gusev, A., Kotliar, D., Ulirsch, J. C., Hormozdiari, F., Nasser, J., O'Connor, L., Van De Geijn, B., et al. (2018). Detecting genome-wide directional effects of transcription factor binding on polygenic disease risk. *Nature genetics*, 50(10):1483.

Vsevolozhskaya, O., Hu, F., and Zaykin, D. (2019). Detecting weak signals by combining small P-values in genetic association studies. *BioRxiv*, page 667238.

Wu, M. C., Lee, S., Cai, T., Li, Y., Boehnke, M., and Lin, X. (2011). Rare-variant association testing for sequencing data with the sequence kernel association test. *Am J Hum Genet*, 89(1):82–93.
