# Supplementary material for: DOT: Gene-set analysis by combining decorrelated association statistics
Source: PLoS Comput Biol. 2020 Apr 14;16(4):e1007819. doi: 10.1371/journal.pcbi.1007819 (PMC7182280; doi:10.1371/journal.pcbi.1007819)
Supplement: Attachment — Submitted filename: response_to_reviewers.pdf [file pcbi.1007819.s003.pdf]

REVIEWERS COMMENTS AND OUR RESPONSES
PCOMPBIOL-D-19-01433 - PLOS Computational Biology
DOT: Gene-set analysis by combining decorrelated association statistics

Thank you for the opportunity to revise this manuscript for the PLOS Computational Biology journal. Below we provide an overview of our responses to reviewer's comments.

Reviewer's 1 comments

Major comments

Overall the authors have addressed my theoretical and methods-related concerns quite well in this revision.

However, I still have serious reservations about the authors' analysis of real data, which analyses a very small set of genes that were not chosen systematically. I previously wrote: "Would it be possible to test a larger set of genes chosen more systematically so that readers can have a sense for whether the authors' approach should in general be preferred over other approaches? Or perhaps to test a few genes chosen by authors of other set testing methods papers?"

The authors did not perform this analysis, and so I still do not know whether their method is more powerful than existing methods beyond the very small set of genes they have analyzed. (The addition in revision of a second phenotype, cleft lip, analyzed in the same way as the first phenotype did not give me a better global sense for why people should use this method.) My understanfing of what the authors have shown is that: a) DOT assigns lower p-values than other methods do to the 4 selected breast cancer genes. This seems weak to me first because lower p-values don't necessarily correspond to higher power (a method can give very low p-values on 1 b) DOT can point at new SNPs associated with breast cancer and cleft lip at these known loci (Tables 10 and 12). But the authors also state (appropriately) that since these results don't come with p-values they should be interpreted with caution, and they also state that cannot conclude that these SNPs are causal but rather only additional proxy SNPs. So I'm unsure what we can confidently learn from these results. I personally don't find (a) or (b) to be strong reasons that practitioners should use DOT.

Overall, I see two ways forward:

1. The authors can carry out a systematic analysis of the performance of their method on real data. For example, they could run the method on a larger set of genes (e.g., all protein coding genes, or all genes expressed in breast tissue, or a set of genes benchmarked in other set testing papers). This would allow the authors to say things like "in a systematic analysis, our method identified X genes to be in loci that are significantly associated with breast cancer, while competing methods identified only Y such genes." I think this would make a much stronger case for the use of this method. And if it's not true, then that is important for potential users to know even if it doesn't preclude publication of the paper.

2. Alternatively, recognizing they have performed extensive revisions already, the authors can add a statement explaining that the genome-wide performance of their method is yet-uncharacterized and would be important to assess in future work.

Thank you very much for this thoughtful suggestion. We are determined to conduct the investigation of genome-wide performance of the proposed method using real data in the future. We added the following to the second from the last paragraph in Discussion:

"An important issue that still remains to be investigated is a systematic analysis of the performance of our method utilizing real genome-wide data. Such analysis would allow one a more thorough assessment of both the type-I error rate, as well as power to detect genetic regions already implicated in susceptibility to disease."

Minor comments

R1.1.1 Just above Table 1, you have a typo: "the column labeled $\hat{\gamma}$ provide the average noncentrality value" ("provide" should be "provides")

This typo is now fixed.

R1.1.2 In the sentence "Different combinations of sample size, the number of causal SNPs, their individual effect sizes and LD patterns among them, resulted in total proportion of phenotypic variance explained...", whose addition I appreciate in this revision, sample size should not be enumerated as one of the parameters that affects the total proportion of phenotypic variance explained.

This sentence is now fixed.

R1.1.3 On page 10, you cite "Min et al. [27, 28]" but neither of refs. 27 or 28 has Min as the last name of a first author in your bibliography.

We have corrected the references as follows:

"We selected four candidate genes (*TOX3, ESR1, FGFR2* and *RAD51B*), for which Shi et al.[1] and O'Brien et al.[2] replicated several previously reported risk SNPs in relation to breast cancer."

R1.1.4 In your response to R1.1.6, you state that eqns 22 and 27 in Reshef et al. 2018 are derived under the null, but this is not true: Eq 22 defines the computation of summary statistics from data (regardless of model) and Equation 27 includes a parameter beta which can be non-zero. A question therefore remains about the relationship between your derivation and the derivation that assumes Gaussian genotypes. (Fine if you want to drop this issue.)

Thank you for this catch. Upon re-examining the derivation in Reshef et al.[3], we now cite their paper and report the relation to our result. We added the following in the revision (a typo, the omitted square in the second term of Eq. 5 has been fixed as well) :

"An alternative derivation of the asymptotic covariance that includes the first two terms of Eq. (5) has been given by Reshef et al.[3], assuming Gaussian genotypes, an assumption justifiable provided that there is a lower bound for minor allele frequency relative to sample size."

References

[1] Shi M, O'Brien KM, Sandler DP, Taylor JA, Zaykin DV, Weinberg CR. Previous GWAS hits in relation to young-onset breast cancer. Breast Cancer Research and Treatment. 2017;161(2):333–344.

[2] O'Brien KM, Shi M, Sandler DP, Taylor JA, Zaykin DV, Keller J, et al. A family-based, genome-wide association study of young-onset breast cancer: inherited variants and maternally mediated effects. European Journal of Human Genetics. 2016;24(9):1316.

[3] Reshef YA, Finucane HK, Kelley DR, Gusev A, Kotliar D, Ulirsch JC, et al. Detecting genome-wide directional effects of transcription factor binding on polygenic disease risk. Nature genetics. 2018;50(10):1483.
